# MISSING IN ACTION: STRATEGIES FOR DEMONSTRATING ORGANIZATIONAL COMMUNICATION IN NURSING HOMES

**DOI:** 10.1093/geroni/igad104.3476

**Published:** 2023-12-21

**Authors:** Patsy Smith, Julie Gordon

**Affiliations:** Fran and Earl Ziegler College of Nursing, The University of Oklahoma Health Sciences Center, Oklahoma City, Oklahoma, United States; Fran and Earl Ziegler College of Nursing, The University of Oklahoma Health Sciences Center, Oklahoma City, Oklahoma, United States

## Abstract

A scoping review was conducted to learn more about strategic organizational communication within long-term care. Strategic organizational communication is a purposeful, system-wide, and system-level effort to transform the organization and delivery of care and services to make it easier to navigate, understand, and coordinate care. Care in nursing homes is complex and must consider residents’ goals, values, and preferences. Communication strategies enacted at the system-level may include direct care staff, human resources, nursing-to-resident and family interactions, and care planning. Strategies at the institutional or system-wide level also include corporate stakeholders, administration, and employee leadership. The aim of the literature search was to locate evidence of communication strategies that reflect corporate and organizational goals, and that exemplify the processes by which communication influences administrative policy. Key words in the search were entered in varying iterations and combinations with the guidance of a skillful research librarian. Key words were long-term care, communication, feedback, nursing homes, patient-centered care, quality improvement. Returns were greater than 2,000, yet seemingly relevant abstracts were fewer than 300, and selected articles were fewer than 30. The research reports reviewed were explanatory or explorative and just one article was found to explore the role of communication strategies and the impact on organizational outcomes. Further, no research reports were found to discuss strategic organizational communication and a combination of resident and organizational outcomes. Rather, most work described specific communication interventions among key groups, many of which lacked sustainable impact. Study results, limitations, and examples of communication strategies are presented herein.

